# Supplementary material for: Targeted, homology-driven gene insertion in stem cells by ZFN-loaded ‘all-in-one’ lentiviral vectors
Source: eLife. 2016 Jun 9;5:e12213. doi: 10.7554/eLife.12213 (PMC4900802; doi:10.7554/eLife.12213)
Supplement: Supplementary file 1. — (A) Sanger sequencing of donor-genome junctions and the AAVS1 locus. The PCR products of 5’ junction and 3’ junction sites as well as the AAVS1 locus from 8 analyzed iPSC clones were Sanger sequenced. The black letters represent genomic DNA at the AAVS1 locus, whereas blue letters represent donor-derived sequences. WT indicates wild-type sequence; indel (+1bp) indicates 1-bp insertion; N.A., not available. (B) Sanger sequencing of PROGNOS-predicted off-target site 1, 2 and 3. The blue sequence indicates the predicted binding site of the right ZFN; the green sequence indicates predicted binding site of the left ZFN; letters highlighted in red indicate mismatches. DOI: http://dx.doi.org/10.7554/eLife.12213.008 [file elife-12213-supp1.docx]

**Supplementary file 1A**

**Sanger sequencing of donor-genome junctions and the *AAVS1* locus.**

| iPSC clone | 5’ junction | 3’ junction | *AAVS1* |
| --- | --- | --- | --- |
| 1 | CCGGAGCACTTCCTTCTCGG | CCTTTCTCTTTCTCCATCCT | WT |
| 2 | CCGGAGCACTTCCTTCTCGG | CCTTTCTCTTTCTCCATCCT | WT |
| 3 | CCGGAGCACTTCCTTCTCGG | CCTTTCTCTTTCTCCATCCT | WT |
| 4 | CCGGAGCACTTCCTTCTCGG | CCTTTCTCTTTCTCCATCCT | WT |
| 5 | CCGGAGCACTTCCTTCTCGG | CCTTTCTCTTTCTCCATCCT | Indel (+1bp) |
| 6 | CCGGAGCACTTCCTTCTCGG | CCTTTCTCTTTCTCCATCCT | WT |
| 7 | CCGGAGCACTTCCTTCTCGG | CCTTTCTCTTTCTCCATCCT | N.A. |
| 8 | CCGGAGCACTTCCTTCTCGG | CCTTTCTCTTTCTCCATCCT | WT |

**Supplementary file 1B**

**Sanger sequencing of PROGNOS-predicted off-target site 1, 2 and 3.**

|  | PROGNOS-predicted off-target site 1 |
| --- | --- |
| iPSC clone | 5’-CTCCTCTGTACCCCCAACTCACCCCACAGTAGATGGCATAGGGACAGGAACTCAAATACAATCTAGATGA-3’  3’-GAGGAGACATGGGGGTTGAGTGGGGTGTCATCTACCGTATCCCTGTCCTTGAGTTTATGTTAGATCTACT-5’ |
| 1 | 5’-CTCCTCTGTACCCCCAACTCACCCCACAGTAGATGGCATAGGGACAGGAACTCAAATACAATCTAGATGA-3’ |
| 2 | 5’-CTCCTCTGTACCCCCAACTCACCCCACAGTAGATGGCATAGGGACAGGAACTCAAATACAATCTAGATGA-3’ |
| 3 | 5’-CTCCTCTGTACCCCCAACTCACCCCACAGTAGATGGCATAGGGACAGGAACTCAAATACAATCTAGATGA-3’ |
| 4 | 5’-CTCCTCTGTACCCCCAACTCACCCCACAGTAGATGGCATAGGGACAGGAACTCAAATACAATCTAGATGA-3’ |
| 5 | 5’-CTCCTCTGTACCCCCAACTCACCCCACAGTAGATGGCATAGGGACAGGAACTCAAATACAATCTAGATGA-3’ |
| 6 | 5’-CTCCTCTGTACCCCCAACTCACCCCACAGTAGATGGCATAGGGACAGGAACTCAAATACAATCTAGATGA-3’ |
| 7 | 5’-CTCCTCTGTACCCCCAACTCACCCCACAGTAGATGGCATAGGGACAGGAACTCAAATACAATCTAGATGA-3’ |
| 8 | 5’-CTCCTCTGTACCCCCAACTCACCCCACAGTAGATGGCATAGGGACAGGAACTCAAATACAATCTAGATGA-3’ |
|  | PROGNOS-predicted off-target site 2 |
| iPSC  clone | 5’-AGAGTTTTAACTTTAGAAGGATCCTATCCAGATAAAAGTAGGGACAGGATGTTCTTTAGGGAATTCCCAG-3’  3’-TCTCAAAATTGAAATCTTCCTAGGATAGGTCTATTTTCATCCCTGTCCTACAAGAAATCCCTTAAGGGTC-5’ |
| 1 | 5’-AGAGTTTTAACTTTAGAAGGATCCTATCCAGATAAAAGTAGGGACAGGATGTTCTTTAGGGAATTCCCAG-3’ |
| 2 | 5’-AGAGTTTTAACTTTAGAAGGATCCTATCCAGATAAAAGTAGGGACAGGATGTTCTTTAGGGAATTCCCAG-3’ |
| 3 | 5’-AGAGTTTTAACTTTAGAAGGATCCTATCCAGATAAAAGTAGGGACAGGATGTTCTTTAGGGAATTCCCAG-3’ |
| 4 | 5’-AGAGTTTTAACTTTAGAAGGATCCTATCCAGATAAAAGTAGGGACAGGATGTTCTTTAGGGAATTCCCAG-3’ |
| 5 | 5’-AGAGTTTTAACTTTAGAAGGATCCTATCCAGATAAAAGTAGGGACAGGATGTTCTTTAGGGAATTCCCAG-3’ |
| 6 | 5’-AGAGTTTTAACTTTAGAAGGATCCTATCCAGATAAAAGTAGGGACAGGATGTTCTTTAGGGAATTCCCAG-3’ |
| 7 | 5’-AGAGTTTTAACTTTAGAAGGATCCTATCCAGATAAAAGTAGGGACAGGATGTTCTTTAGGGAATTCCCAG-3’ |
| 8 | 5’-AGAGTTTTAACTTTAGAAGGATCCTATCCAGATAAAAGTAGGGACAGGATGTTCTTTAGGGAATTCCCAG-3’ |
|  | PROGNOS-predicted off-target site 3 |
| iPSC clone | 5’-CTGGGGGGTCGCCAGTGGTGACCCCGCAGTGGGTGCGACACTGGGGGGTTGCCAGTGGTGACCACAGGA-3’  3’-GACCCCCCAGCGGTCACCACTGGGGCGTCACCCACGCTGTGACCCCCCAACGGTCACCACTGGTGTCCT-5’ |
| 1 | 5’-CTGGGGGGTCGCCAGTGGTGACCCCGCAGTGGGTGCGACACTGGGGGGTTGCCAGTGGTGACCACAGGA-3’ |
| 2 | 5’-CTGGGGGGTCGCCAGTGGTGACCCCGCAGTGGGTGCGACACTGGGGGGTTGCCAGTGGTGACCACAGGA-3’ |
| 3 | 5’-CTGGGGGGTCGCCAGTGGTGACCCCGCAGTGGGTGCGACACTGGGGGGTTGCCAGTGGTGACCACAGGA-3’ |
| 4 | 5’-CTGGGGGGTCGCCAGTGGTGACCCCGCAGTGGGTGCGACACTGGGGGGTTGCCAGTGGTGACCACAGGA-3’ |
| 5 | 5’-CTGGGGGGTCGCCAGTGGTGACCCCGCAGTGGGTGCGACACTGGGGGGTTGCCAGTGGTGACCACAGGA-3’ |
| 6 | 5’-CTGGGGGGTCGCCAGTGGTGACCCCGCAGTGGGTGCGACACTGGGGGGTTGCCAGTGGTGACCACAGGA-3’ |
| 7 | 5’-CTGGGGGGTCGCCAGTGGTGACCCCGCAGTGGGTGCGACACTGGGGGGTTGCCAGTGGTGACCACAGGA-3’ |
| 8 | 5’-CTGGGGGGTCGCCAGTGGTGACCCCGCAGTGGGTGCGACACTGGGGGGTTGCCAGTGGTGACCACAGGA-3’ |
