# Supplementary material for: Targeted, homology-driven gene insertion in stem cells by ZFN-loaded ‘all-in-one’ lentiviral vectors
Source: eLife. 2016 Jun 9;5:e12213. doi: 10.7554/eLife.12213 (PMC4900802; doi:10.7554/eLife.12213)
Supplement: Supplementary file 2. — (A) Primers used for molecular cloning and PCR amplification of junction sites. (B) Primers used to amplify PROGNOS-predicted off-target sites. DOI: http://dx.doi.org/10.7554/eLife.12213.009 [file elife-12213-supp2.docx]

**Supplementary file 2A**

**Primers used for molecular cloning and PCR amplification of junction sites.**

| Primer names | Sequence (5’-3’) |
| --- | --- |
| YJ205R | AGATAGCACTGGGGACTCTTTAAG |
| YJ208R | AAGGTGTTCAGGAGAAGGACAATG |
| YJ210R | TGACGTCGACTACCGGGTAGGGGAGGCGCT |
| YJ219F | TGACCGTACGTGTTTTATCGGTCTGTATAT |
| YJ222F | TTCGGGTCACCTCTCACTCC |
| YJ223R | GGCTCCATCGTAAGCAAACC |
| YJ224F | AAAACAGTTTGCATTCATGGAGGGC |
| YJ225R | AGAAGCCTATAAAATAGAGCCCTGT |
| YJ251F | TCGAAAGGCCCGGAGATGAGGAAGA |
| YJ252F | AATGTGTGCGAGGCCAGAGGCCACT |
| YJ253R | CCAACCTGTTAGAGCTACTGCAATT |
| YJ262F | TGACTGTACATGTTTTATCGGTCTGTATATCGAGGTTTAT |
| YJ599F | AGTCCCCAGTGCTATCTGGG |
| YJ600R | AGAAACACAGCCCCAGGTGG |
| YJ601F | ATATTCCTCCGCCCAGAGCA |
| YJ602R | GCCGGGAATCAAGAGTCACC |
| YJ666R | TAAGGAAAGAAGGATGGAGA |
| YJ706F | CGTCTCTCTCCTGAGTCCGG |
| YJ707F | CTGGGTCACCTCTACGGCTG |
| YJ738R | TCCCCCGGGCTGCAGGAATT |
| YJ825F | TGAGGTGAGAGGATTGCTTG |
| YJ833F | TGCAGTGAGCCATGATCGTG |
| 0279 | GATCTCTCGTGGGATCATTG |
| AgeI-int(s) | TGTACACATTTAGAAGGAAA |
| int-D64V(as) | TTTCCTTCTAAATGTGTACAAACTAGCTGCCATATTCCTGGGC |
| int-D64V(s) | TGTACACATTTAGAAGGAAA |
| AccIII-int(as) | TAGCTCCGGAATTCCATGTGTTAA |
| Puro-1 | CCGAGTACAAGCCCACGGTG |
| Puro-2 | CAAGAGAAAAACAATGATCCC |

**Supplementary file 2B**

**Primers used to amplify PROGNOS-predicted off-target sites.**

| On- and off-targets | Chromosomal Coordinates | Primer  names | Sequence (5’-3’) |
| --- | --- | --- | --- |
| AAVS1 On-target | chr19:55627120-55627149 | YJ840F | GGTGGGGGTTAGACCCAATA |
|  |  | YJ841R | TGGGACCACCTTATATTCCC |
| AAVS1 Off-target 1 | chr13:40779943-40779972 | YJ842F | TGATCTGCTGCTGGACTTGA |
|  |  | YJ843R | CTGTGAGGATGTCATCTGTG |
| AAVS1 Off-target 2 | chr4:153500533-153500562 | YJ844F | TGCAGATACTCCGCTCTTTG |
|  |  | YJ845R | GCTTTGGCGTGTATATGTAT |
| AAVS1 Off-target 3 | chrX:153791980-153792008 | YJ846F | CGGCCAGGTGGGCCTCTGAG |
|  |  | YJ847R | GGAGAGGGACCACAGTGAGC |
| AAVS1 Off-target 4 | chrX:153868919-153868947 | YJ848F | GGAGAGGGACCACAGTGAGC |
|  |  | YJ849R | CGGCCAGGTGGGCCTCTGAG |
| AAVS1 Off-target 5 | chr10:44698021-44698049 | YJ850F | TGTGGTATTCTGTTACAGAA |
|  |  | YJ851R | CTCAGGGGAGAGGTAATGAG |
| AAVS1 Off-target 6 | chr11:130345617-130345646 | YJ852F | GCGGGGGCAGAGGGGGTGCC |
|  |  | YJ853R | GGGCGCAGTGCTGTCGTCAG |
| AAVS1 Off-target 7 | chr9:139233282-139233310 | YJ856F | GGCTGAATTACCGCGGCCCA |
|  |  | YJ857R | TGGGAGGGGGACCCCACCCG |
| CCR5 On-target | chr3:46414544-46414572 | YJ888F | GTGAAGCAAATCGCAGCCCG |
|  |  | YJ889R | AGAAGGAAAAACAGGTCAGA |
| CCR5 Off-target 1 | chr4:8114476-8114504 | YJ862F | CACAACAGCCACGGTCCCAG |
|  |  | YJ863R | GGGAGGAGGAGCCTCGGACA |
| CCR5 Off-target 2 | chr14:88239007-88239036 | YJ884F | CGAATCTTCTATTGGTGAAA |
|  |  | YJ885R | ATAGGAACACATTTCTTTTC |
| CCR5 Off-target 3 | chr3:46399205-46399233 | YJ890F | TGAAGCAAATTGGGGCCCAA |
|  |  | YJ891R | ATAAGAAAAAGCAGATCAGA |
| CCR5 Off-target 4 | chr9:12916961-12916990 | YJ868F | AGGCTATATTCCCAGCACAT |
|  |  | YJ869R | AATATTAGGCACATTTTCTC |
| CCR5 Off-target 5 | chr21:33398096-33398125 | YJ870F | GCTAAGACCTATGGTAAAGA |
|  |  | YJ871R | ATCTTCCACCCACAAATTTA |
| CCR5 Off-target 6 | chr8:78205879-78205908 | YJ872F | AAGGTTGCTACTATTTACAG |
|  |  | YJ873R | ATGTCTTCCACCCACATATT |
| CCR5 Off-target 7 | chr17:67106159-67106188 | YJ874F | GCTAAGATCTAACGTAAGAA |
|  |  | YJ875R | ATGTCTTCCACCTACACACT |
| CCR5 Off-target 8 | chr5:154370244-154370272 | YJ876F | GAAAGGAAGCAGGGGAGAAT |
|  |  | YJ877R | GCTGCCTTCCTCGTCACCTC |
| CCR5 Off-target 9 | chr10:47003744-47003772 | YJ878F | AGTTTGAATCTGGATATATA |
|  |  | YJ879R | ACTCAGTGCCAGGCACTACT |
