# Supplementary material for: Targeted, homology-driven gene insertion in stem cells by ZFN-loaded ‘all-in-one’ lentiviral vectors
Source: eLife. 2016 Jun 9;5:e12213. doi: 10.7554/eLife.12213 (PMC4900802; doi:10.7554/eLife.12213)
Supplement: Supplementary file 3. — Homology arms and transgene expression cassettes in pLV/AAVS1-donor-egfp-PGK, pLV/AAVS1-donor-fluc-PGK, pLV/AAVS1-donor-puro-PGK and pLV/CCR5-donor-puro-PGK are provided. For each transgene expression cassette, sequences are annotated as listed below the sequence and indicated with different colors. DOI: http://dx.doi.org/10.7554/eLife.12213.010 [file elife-12213-supp3.docx]

**Supplementary file 3 - Donor sequences**

**Homology arms and transgene expression cassette in pLV/AAVS1-donor-egfp-PGK**

tccttctcggcgctgcaccacgtgatgtcctctgagcggatcctccccgtgtctgggtcctctccgggcatctctcctccctcacccaaccccatgccgtcttcactcgctgggttcccttttccttctccttctggggcctgtgccatctctcgtttcttaggatggccttctccgacggatgtctcccttgcgtcccgcctccccttcttgtaggcctgcatcatcaccgtttttctggacaaccccaaagtaccccgtctccctggctttagccacctctccatcctcttgctttctttgcctggacaccccgttctcctgtggattcgggtcacctctcactcctttcatttgggcagctcccctaccccccttacctctctagtctgtgctagctcttccagccccctgtcatggcatcttccaggggtccgagagctcagctagtcttcttcctccaacccgggcccctatgtccacttcaggacagcatgtttgctgcctccagggatcctgtgtccccgagctgggaccaccttatattcccagggccggttaatgtggctctggttctgggtacttttatctgtcccctccaccggttctagacgtacgtgttttatcggtctgtatatcgaggtttatttattaatttgaatagatattaagttttattatatttacacttacatactaataataaattcaacaaacaatttatttatgtttatttatttattaaaaaaaacaaaaactcaaaatttcttctataaagtaacaaaacttttatcgaattcctgcagcccgggggatccactagttctagagcggccgctagctcgaccagcttctgatggaattagaacttggcaaaacaatactgagaatgaagtgtatgtggaacagaggctgctgatctcgttcttcaggctatgaaactgacacatttggaaaccacagtacttagaaccacaaagtgggaatcaagagaaaaacaatgatcccacgagagatctatagatctatagatcatgagtgggaggaatgagctggcccttaatttggttttgcttgtttaaattatgatatccaactatgaaacattatcataaagcaatagtaaagagccttcagtaaagagcaggcatttatctaatcccaccccacccccacccccgtagctccaatccttccattcaaaatgtaggtactctgttctcacccttcttaacaaagtatgacaggaaaaacttccattttagtggacatctttattgtttaatagatcatcaatttctgcatcccggggatctgatatcatcgatgcatggggtcgtgcgctcctttcggtcgggcgctgcgggtcgtggggcgggcgttacttgtacagctcgtccatgccgagagtgatcccggcggcggtcacgaactccagcaggaccatgtgatcgcgcttctcgttggggtctttgctcagggcggactgggtgctcaggtagtggttgtcgggcagcagcacggggccgtcgccgatgggggtgttctgctggtagtggtcggcgagctgcacgctgccgtcctcgatgttgtggcggatcttgaagttcaccttgatgccgttcttctgcttgtcggccatgatatagacgttgtggctgttgtagttgtactccagcttgtgccccaggatgttgccgtcctccttgaagtcgatgcccttcagctcgatgcggttcaccagggtgtcgccctcgaacttcacctcggcgcgggtcttgtagttgccgtcgtccttgaagaagatggtgcgctcctggacgtagccttcgggcatggcggacttgaagaagtcgtgctgcttcatgtggtcggggtagcggctgaagcactgcacgccgtaggtcagggtggtcacgagggtgggccagggcacgggcagcttgccggtggtgcagatgaacttcagggtcagcttgccgtaggtggcatcgccctcgccctcgccggacacgctgaacttgtggccgtttacgtcgccgtccagctcgaccaggatgggcaccaccccggtgaacagctcctcgcccttgctcaccatggtggcgaccggtggatcccgctagaggtcgaaaggcccggagatgaggaagaggagaacagcgcggcagacgtgcgcttttgaagcgtgcagaatgccgggcctccggaggaccttcgggcgcccgccccgcccctgagcccgcccctgagcccgcccccggacccaccccttcccagcctctgagcccagaaagcgaaggagcaaagctgctattggccgctgccccaaaggcctacccgcttccattgctcagcggtgctgtccatctgcacgagactagtgagacgtgctacttccatttgtcacgtcctgcacgacgcgagctgcggggcgggggggaacttcctgactaggggaggagtagaaggtggcgcgaaggggccaccaaagaacggagccggttggcgcctaccggtggatgtggaatgtgtgcgaggccagaggccacttgtgtagcgccaagtgcccagcggggctgctaaagcgcatgctccagactgccttgggaaaagcgcctcccctacccggtagtcgactggtgacagaaaagccccatccttaggcctcctccttcctagtctcctgatattgggtctaacccccacctcctgttaggcagattccttatctggtgacacacccccatttcctggagccatctctctccttgccagaacctctaaggtttgcttacgatggagccagagaggatcctgggagggagagcttggcagggggtgggagggaagggggggatgcgtgacctgcccggttctcagtggccaccctgcgctaccctctcccagaacctgagctgctctgacgcggccgtctggtgcgtttcactgatcctggtgctgcagcttccttacacttcccaagaggagaagcagtttggaaaaacaaaatcagaataagttggtcctgagttctaactttggctcttcacctttctagtccccaatttatattgttcctccgtgcgtcagttttacctgtgagataaggccagtagccagccccgtcctggcagggctgtggtgaggaggggggtgtccgtgtggaaaactccctttgtgagaatggtgcgtcctaggtgttcaccaggtcgtggccgcctctactccctttctctt

**Homology arm; poly(A) containing region; eGFP gene; PGK promoter**

**Homology arms and transgene expression cassette in pLV/AAVS1-donor-fluc-PGK**

tccttctcggcgctgcaccacgtgatgtcctctgagcggatcctccccgtgtctgggtcctctccgggcatctctcctccctcacccaaccccatgccgtcttcactcgctgggttcccttttccttctccttctggggcctgtgccatctctcgtttcttaggatggccttctccgacggatgtctcccttgcgtcccgcctccccttcttgtaggcctgcatcatcaccgtttttctggacaaccccaaagtaccccgtctccctggctttagccacctctccatcctcttgctttctttgcctggacaccccgttctcctgtggattcgggtcacctctcactcctttcatttgggcagctcccctaccccccttacctctctagtctgtgctagctcttccagccccctgtcatggcatcttccaggggtccgagagctcagctagtcttcttcctccaacccgggcccctatgtccacttcaggacagcatgtttgctgcctccagggatcctgtgtccccgagctgggaccaccttatattcccagggccggttaatgtggctctggttctgggtacttttatctgtcccctccaccggttctagacgtacgtgttttatcggtctgtatatcgaggtttatttattaatttgaatagatattaagttttattatatttacacttacatactaataataaattcaacaaacaatttatttatgtttatttatttattaaaaaaaacaaaaactcaaaatttcttctataaagtaacaaaacttttatcgaattcctgcagcccgggggatccactagttctagagcggccgctagctcgaccagcttctgatggaattagaacttggcaaaacaatactgagaatgaagtgtatgtggaacagaggctgctgatctcgttcttcaggctatgaaactgacacatttggaaaccacagtacttagaaccacaaagtgggaatcaagagaaaaacaatgatcccacgagagatctatagatctatagatcatgagtgggaggaatgagctggcccttaatttggttttgcttgtttaaattatgatatccaactatgaaacattatcataaagcaatagtaaagagccttcagtaaagagcaggcatttatctaatcccaccccacccccacccccgtagctccaatccttccattcaaaatgtaggtactctgttctcacccttcttaacaaagtatgacaggaaaaacttccattttagtggacatctttattgtttaatagatcatcaatttctgcatcccggggatctgatatcatcgatgcatggggtcgtgcgctcctttcggtcgggcgctgcgggtcgtggggcgggcgttacacggcgatctttccgcccttcttggcctttatgaggatctctctgatttttcttgcgtcgagttttccggtaagacctttcggtacttcgtccacaaacacaactcctccgcgcaactttttcgcggttgttacttgactggcgacgtaatccacgatctctttttccgtcatcgtctttccgtgctccaaaacaacaacggcggcgggaagttcaccggcgtcatcgtcgggaagacctgcgacacctgcgtcgaagatgttggggtgttggagcaagatggattccaattcagcgggagccacctgatagcctttgtacttaatcagagacttcaggcggtcaacgatgaagaagtgttcgtcttcgtcccagtaagctatgtctccagaatgtagccatccatccttgtcaatcaaggcgttggtcgcttccggattgtttacataaccggacataatcataggacctctcacacacagttcgcctctttgattaacgcccagcgttttcccggtatccagatccacaaccttcgcttcaaaaaatggaacaactttaccgaccgcgcccggtttatcatccccctcgggtgtaatcagaatagctgatgtagtctcagtgagcccatatccttgcctgatacctggcagatggaacctcttggcaaccgcttccccgacttccttagagaggggagcgccaccagaagcaatttcgtgtaaattagataaatcgtatttgtcaatcagagtgcttttggcgaagaaggagaatagggttggcaccagcagcgcactttgaatcttgtaatcctgaaggctcctcagaaacagctcttcttcaaatctatacattaagacgactcgaaatccacatatcaaatatccgagtgtagtaaacattccaaaaccgtgatggaatggaacaacacttaaaatcgcagtatccggaatgatttgattgccaaaaataggatctctggcatgcgagaatctcacgcaggcagttctatgaggcagagcgacacctttaggcagaccagtagatccagaggagttcatgatcagtgcaattgtcttgtccctatcgaaggactctggcacaaaatcgtattcattaaaaccgggaggtagatgagatgtgacgaacgtgtacatcgactgaaatccctggtaatccgttttagaatccatgataataattttttggatgattgggagctttttttgcacgttcaaaattttttgcaacccctttttggaaacgaacaccacggtaggctgcgaaatgcccatactgttgagcaattcacgttcattataaatgtcgttcgcgggcgcaactgcaactccgataaataacgcgcccaacaccggcataaagaattgaagagagttttcactgcatacgacgattctgtgatttgtattcagcccatatcgtttcatagcttctgccaaccgaacggacatttcgaagtactcagcgtaagtgatgtccacctcgatatgtgcatctgtaaaagcaattgttccaggaaccagggcgtatctcttcatagccttatgcagttgctctccagcggttccatcttccagcggatagaatggcgccgggcctttctttatgtttttggcgtcttccatggatcccgctagaggtcgaaaggcccggagatcgaaaggcccggagatgaggaagaggagaacagcgcggcagacgtgcgcttttgaagcgtgcagaatgccgggcctccggaggaccttcgggcgcccgccccgcccctgagcccgcccctgagcccgcccccggacccaccccttcccagcctctgagcccagaaagcgaaggagcaaagctgctattggccgctgccccaaaggcctacccgcttccattgctcagcggtgctgtccatctgcacgagactagtgagacgtgctacttccatttgtcacgtcctgcacgacgcgagctgcggggcgggggggaacttcctgactaggggaggagtagaaggtggcgcgaaggggccaccaaagaacggagccggttggcgcctaccggtggatgtggaatgtgtgcgaggccagaggccacttgtgtagcgccaagtgcccagcggggctgctaaagcgcatgctccagactgccttgggaaaagcgcctcccctacccggtagtcgactggtgacagaaaagccccatccttaggcctcctccttcctagtctcctgatattgggtctaacccccacctcctgttaggcagattccttatctggtgacacacccccatttcctggagccatctctctccttgccagaacctctaaggtttgcttacgatggagccagagaggatcctgggagggagagcttggcagggggtgggagggaagggggggatgcgtgacctgcccggttctcagtggccaccctgcgctaccctctcccagaacctgagctgctctgacgcggccgtctggtgcgtttcactgatcctggtgctgcagcttccttacacttcccaagaggagaagcagtttggaaaaacaaaatcagaataagttggtcctgagttctaactttggctcttcacctttctagtccccaatttatattgttcctccgtgcgtcagttttacctgtgagataaggccagtagccagccccgtcctggcagggctgtggtgaggaggggggtgtccgtgtggaaaactccctttgtgagaatggtgcgtcctaggtgttcaccaggtcgtggccgcctctactccctttctctt

**Homology arm; poly(A) containing region; firefly luciferase gene; PGK promoter**

**Homology arms and transgene expression cassette in pLV/AAVS1-donor-puro-PGK**

tccttctcggcgctgcaccacgtgatgtcctctgagcggatcctccccgtgtctgggtcctctccgggcatctctcctccctcacccaaccccatgccgtcttcactcgctgggttcccttttccttctccttctggggcctgtgccatctctcgtttcttaggatggccttctccgacggatgtctcccttgcgtcccgcctccccttcttgtaggcctgcatcatcaccgtttttctggacaaccccaaagtaccccgtctccctggctttagccacctctccatcctcttgctttctttgcctggacaccccgttctcctgtggattcgggtcacctctcactcctttcatttgggcagctcccctaccccccttacctctctagtctgtgctagctcttccagccccctgtcatggcatcttccaggggtccgagagctcagctagtcttcttcctccaacccgggcccctatgtccacttcaggacagcatgtttgctgcctccagggatcctgtgtccccgagctgggaccaccttatattcccagggccggttaatgtggctctggttctgggtacttttatctgtcccctccaccggttctagacgtacgtgttttatcggtctgtatatcgaggtttatttattaatttgaatagatattaagttttattatatttacacttacatactaataataaattcaacaaacaatttatttatgtttatttatttattaaaaaaaacaaaaactcaaaatttcttctataaagtaacaaaacttttatcgaattcctgcagcccgggggatccactagttctagagcggccgctagctcgaccagcttctgatggaattagaacttggcaaaacaatactgagaatgaagtgtatgtggaacagaggctgctgatctcgttcttcaggctatgaaactgacacatttggaaaccacagtacttagaaccacaaagtgggaatcaagagaaaaacaatgatcccacgagagatctatagatctatagatcatgagtgggaggaatgagctggcccttaatttggttttgcttgtttaaattatgatatccaactatgaaacattatcataaagcaatagtaaagagccttcagtaaagagcaggcatttatctaatcccaccccacccccacccccgtagctccaatccttccattcaaaatgtaggtactctgttctcacccttcttaacaaagtatgacaggaaaaacttccattttagtggacatctttattgtttaatagatcatcaatttctgcatcccggggatctgatatcatcgatgcatggggtcgtgcgctcctttcggtcgggcgctgcgggtcgtggggcgggcgtcaggcaccgggcttgcgggtcatgcaccaggtgcgcggtccttcgggcacctcgacgtcggcggtgacggtgaagccgagccgctcgtagaaggggaggttgcggggcgcggaggtctccaggaaggcgggcaccccggcgcgctcggccgcctccactccggggagcacgacggcgctgcccagacccttgccctggtggtcgggcgagacgccgacggtggccaggaaccacgcgggctccttgggccggtgcggcgccaggaggccttccatctgttgctgcgcggccagccgggaaccgctcaactcggccatgcgcgggccgatctcggcgaacaccgcccccgcttcgacgctctccggcgtggtccagaccgccaccgcggcgccgtcgtccgcgacccacaccttgccgatgtcgagcccgacgcgcgtgaggaagagttcttgcagctcggtgacccgctcgatgtggcggtccggatcgacggtgtggcgcgtggcggggtagtcggcgaacgcggcggcgagggtgcgtacggcccgggggacgtcgtcgcgggtggcgaggcgcaccgtgggcttgtactcggtcatggtaagcttcagctgctcgagatctagatggatgcaggtcgaaaggcccggagatgaggaagaggagaacagcgcggcagacgtgcgcttttgaagcgtgcagaatgccgggcctccggaggaccttcgggcgcccgccccgcccctgagcccgcccctgagcccgcccccggacccaccccttcccagcctctgagcccagaaagcgaaggagcaaagctgctattggccgctgccccaaaggcctacccgcttccattgctcagcggtgctgtccatctgcacgagactagtgagacgtgctacttccatttgtcacgtcctgcacgacgcgagctgcggggcgggggggaacttcctgactaggggaggagtagaaggtggcgcgaaggggccaccaaagaacggagccggttggcgcctaccggtggatgtggaatgtgtgcgaggccagaggccacttgtgtagcgccaagtgcccagcggggctgctaaagcgcatgctccagactgccttgggaaaagcgcctcccctacccggtagtcgactggtgacagaaaagccccatccttaggcctcctccttcctagtctcctgatattgggtctaacccccacctcctgttaggcagattccttatctggtgacacacccccatttcctggagccatctctctccttgccagaacctctaaggtttgcttacgatggagccagagaggatcctgggagggagagcttggcagggggtgggagggaagggggggatgcgtgacctgcccggttctcagtggccaccctgcgctaccctctcccagaacctgagctgctctgacgcggccgtctggtgcgtttcactgatcctggtgctgcagcttccttacacttcccaagaggagaagcagtttggaaaaacaaaatcagaataagttggtcctgagttctaactttggctcttcacctttctagtccccaatttatattgttcctccgtgcgtcagttttacctgtgagataaggccagtagccagccccgtcctggcagggctgtggtgaggaggggggtgtccgtgtggaaaactccctttgtgagaatggtgcgtcctaggtgttcaccaggtcgtggccgcctctactccctttctctt

**Homology arm; poly(A) containing region; puromycin resistance gene;**

**PGK promoter**

**Homology arms and transgene expression cassette in pLV/CCR5-donor-puro-PGK**

GCCTGGGCGACAGAGTGAGACCCTGTCTCACAACAACAACAGCAACAAAAAGGCTGAGCTGCACCATGCTTGACCCAGTTTCTTAAAATTGTTGTCAAAGCTTCATTCACTCCATGGTGCTATAGAGCACAAGATTTTATTTGGTGAGATGGTGCTTTCATGAATTCCCCCAACAGAGCCAAGCTCTCCATCTAGTGGACAGGGAAGCTAGCAGCAAACCTTCCCTTCACTACAAAACTTCATTGCTTGGCCAAAAAGAGAGTTAATTCAATGTAGACATCTATGTAGGCAATTAAAAACCTATTGATGTATAAAACAGTTTGCATTCATGGAGGGCAACTAAATACATTCTAGGACTTTATAAAAGATCACTTTTTATTTATGCACAGGGTGGAACAAGatggattatcaagtgtcaagtccaatctatgacatcaattattatacatcggagccctgccaaaaaatcaatgtgaagcaaatcgcagcccgcctcctgcctccgctctactcactggtgttcatctttggttttgtgggcaacatgctgaccggttctagacgtacgtgttttatcggtctgtatatcgaggtttatttattaatttgaatagatattaagttttattatatttacacttacatactaataataaattcaacaaacaatttatttatgtttatttatttattaaaaaaaacaaaaactcaaaatttcttctataaagtaacaaaacttttatcgaattcctgcagcccgggggatccactagttctagagcggccgctagctcgaccagcttctgatggaattagaacttggcaaaacaatactgagaatgaagtgtatgtggaacagaggctgctgatctcgttcttcaggctatgaaactgacacatttggaaaccacagtacttagaaccacaaagtgggaatcaagagaaaaacaatgatcccacgagagatctatagatctatagatcatgagtgggaggaatgagctggcccttaatttggttttgcttgtttaaattatgatatccaactatgaaacattatcataaagcaatagtaaagagccttcagtaaagagcaggcatttatctaatcccaccccacccccacccccgtagctccaatccttccattcaaaatgtaggtactctgttctcacccttcttaacaaagtatgacaggaaaaacttccattttagtggacatctttattgtttaatagatcatcaatttctgcatcccggggatctgatatcatcgatgcatggggtcgtgcgctcctttcggtcgggcgctgcgggtcgtggggcgggcgtcaggcaccgggcttgcgggtcatgcaccaggtgcgcggtccttcgggcacctcgacgtcggcggtgacggtgaagccgagccgctcgtagaaggggaggttgcggggcgcggaggtctccaggaaggcgggcaccccggcgcgctcggccgcctccactccggggagcacgacggcgctgcccagacccttgccctggtggtcgggcgagacgccgacggtggccaggaaccacgcgggctccttgggccggtgcggcgccaggaggccttccatctgttgctgcgcggccagccgggaaccgctcaactcggccatgcgcgggccgatctcggcgaacaccgcccccgcttcgacgctctccggcgtggtccagaccgccaccgcggcgccgtcgtccgcgacccacaccttgccgatgtcgagcccgacgcgcgtgaggaagagttcttgcagctcggtgacccgctcgatgtggcggtccggatcgacggtgtggcgcgtggcggggtagtcggcgaacgcggcggcgagggtgcgtacggcccgggggacgtcgtcgcgggtggcgaggcgcaccgtgggcttgtactcggtcatggtaagcttcagctgctcgagatctagatggatgcaggtcgaaaggcccggagatgaggaagaggagaacagcgcggcagacgtgcgcttttgaagcgtgcagaatgccgggcctccggaggaccttcgggcgcccgccccgcccctgagcccgcccctgagcccgcccccggacccaccccttcccagcctctgagcccagaaagcgaaggagcaaagctgctattggccgctgccccaaaggcctacccgcttccattgctcagcggtgctgtccatctgcacgagactagtgagacgtgctacttccatttgtcacgtcctgcacgacgcgagctgcggggcgggggggaacttcctgactaggggaggagtagaaggtggcgcgaaggggccaccaaagaacggagccggttggcgcctaccggtggatgtggaatgtgtgcgaggccagaggccacttgtgtagcgccaagtgcccagcggggctgctaaagcgcatgctccagactgccttgggaaaagcgcctcccctacccggtagtcgacgctgaagagcatgactgacatctacctgctcaacctggccatctctgacctgtttttccttcttactgtccccttctgggctcactatgctgccgcccagtgggactttggaaatacaatgtgtcaactcttgacagggctctattttataggcttcttctctggaatcttcttcatcatcctcctgacaatcgataggtacctggctgtcgtccatgctgtgtttgctttaaaagccaggacggtcacctttggggtggtgacaagtgtgatcacttgggtggtggctgtgtttgcgtctctcccaggaatcatctttaccagatctcaaaaagaaggtcttcattacacctgcagctctcattttccatacagtcagtatcaattctggaagaatttccagacattaaagatagtcatcttggggctggtcctgccgctgcttgtcatggtcatctgctactcgggaatcctaaaaactctgcttcggtgtcgaaatgagaagaagaggcacagggctgtgaggcttatcttcaccatcatgattgtt

**Homology arm; poly(A) containing region; puromycin resistance gene;**

**PGK promoter**
